# A novel truncating variant of *SPAST* associated with hereditary spastic paraplegia indicates a haploinsufficiency pathogenic mechanism

**DOI:** 10.3389/fneur.2022.1005544

**Published:** 2022-11-14

**Authors:** Haitian Nan, Min Chu, Li Liu, Kexin Xie, Liyong Wu

**Affiliations:** Department of Neurology, Xuanwu Hospital, Capital Medical University, Beijing, China

**Keywords:** hereditary spastic paraplegias, SPG4, spastin, *SPAST*, haploinsufficiency

## Abstract

**Introduction:**

Hereditary spastic paraplegias (HSPs) are genetic neurodegenerative diseases. The most common form of pure HSP that is inherited in an autosomal dominant manner is spastic paraplegia type 4 (SPG4), which is caused by mutations in the *SPAST* gene. Different theories have been proposed as the mechanism underlying *SPAST*-HSP for different types of genetic mutations, including gain- and loss-of-function mechanisms. To better understand the mutation mechanisms, we performed genetic analysis and investigated a truncating *SPAST* variant that segregated with disease in one family.

**Objectives and methods:**

We described a pure HSP pedigree with family members across four generations. We performed genetic analysis and investigated a novel frameshift pathogenic variant (c.862_863dupAC, p. H289Lfs^*^27) in this family. We performed reverse transcription-polymerase chain reaction (RT-PCR), Sanger sequencing, and quantitative RT-PCR using total RNA from an Epstein-Barr virus-induced lymphoblastoid cell line produced from the proband. We also performed Western blotting on cell lysates to investigate if the protein expression of spastin is affected by this variant.

**Results:**

This variant (c.862_863dupAC, p. H289Lfs^*^27) co-segregated with pure HSP in this family and is not registered in any public database. Measurement of *SPAST* transcripts in lymphoblasts from the proband demonstrated a reduction of *SPAST* transcript levels through likely nonsense-mediated mRNA decay. Immunoblot analyses demonstrated a reduction of spastin protein expression levels in lymphoblasts.

**Conclusion:**

We report an SPG4 family with a novel heterozygous frameshift variant p.H289Lfs^*^27 in *SPAST*. Our study implies haploinsufficiency as the pathogenic mechanism for this variant and expands the known mutation spectrum of *SPAST*.

## Introduction

Although all related to axonal degeneration in the pyramidal tract, hereditary spastic paraplegias (HSPs) are clinically and genetically heterogeneous neurodegenerative disorders that cause progressive weakening and spasticity in the lower limbs (1, 2). The age of onset varies from early childhood to 70 years of age, and they can be inherited via autosomal dominant (AD), autosomal recessive (AR), X-linked, or mitochondrial mechanisms (3). Pure HSP is defined as a mostly isolated pyramidal syndrome that mostly affects the lower limbs, either with or without vibration sensation impairment and urine urgency, while complicated HSP has a more complex clinical presentation and additional neurological abnormalities (2).

**Figure 1:**
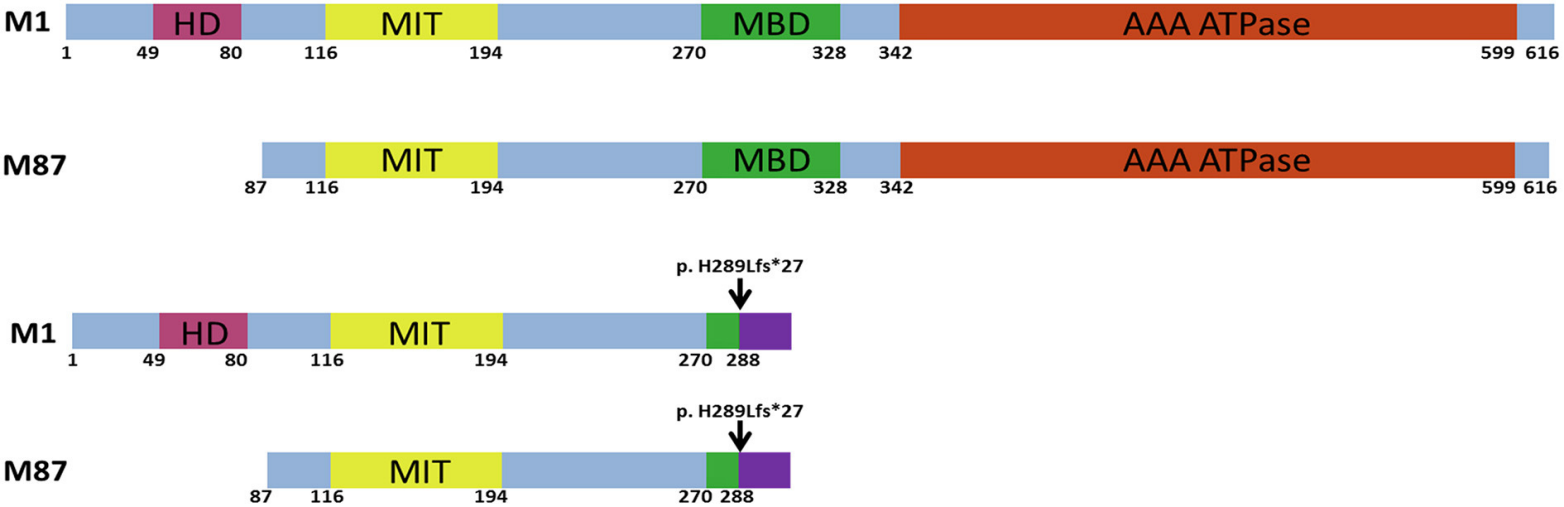
Schematic structure of the human wild-type and mutant spastin proteins. M1 and M87 spastin isoforms are presented. HD, hydrophobic domain; MIT, microtubule interacting and transport domain; MBD, microtubule-binding domain; AAA ATPase, ATPase associated with diverse cellular activities domain. The black arrow indicates the location of the c.862_863dupAC frameshift variant.

The most common form of pure HSP is called spastic paraplegia type 4 (SPG4), which is caused by a heterozygous mutation of *SPAST* and is identified in over 25% of HSP cases (4, 5). Most of the time, SPG4 is regarded as a pure HSP (6). Spastin, a microtubule-severing AAA ATPase that is encoded by *SPAST*, controls microtubule dynamics and is crucial for cell division and neurogenesis (Figure 1) (7). Spastin transcript is widely expressed in non-neuronal tissues and the nervous system (8), but the disease phenotype is remarkably restricted to corticospinal axons (9). It is still debatable whether the toxic gain-of-function characteristics of the mutant spastin proteins or haploinsufficiency is the cause of the disease (6). We report a novel frameshift pathogenic variant in *SPAST* that co-segregated with a pure phenotype of HSP in a four-generation family. Our data suggest that the c.862_863dupAC (p. H289Lfs^*^27) variant exerts its pathogenic effect by haploinsufficiency.

## Materials and methods

### Whole-exome sequencing study

The genomic DNA was isolated from peripheral blood leukocytes (QIAamp DNA Blood Kits, QIAGEN). Exome capture was performed with a SureSelect Human All Exon V6+UTR (89Mb) Kit (Agilent Technologies, Santa Clara, CA, USA). Paired-end sequencing was carried out on a HiSeq2500 (Illumina, San Diego, CA, USA) using a HiSeq SBS Kit V4 (Illumina), which generated 100-bp reads. The average and minimum sequencing depths were 125× and 20× , respectively. The reference databases utilized included hg38 (GRCh38) (http://genome.ucsc.edu), HGMD (https://portal.biobase-international.com), gnomAD (http://gnomad.broadinstitute.org), ClinVar (https://www.ncbi.nlm.nih.gov/clinvar/), and dbSNP (https://www.ncbi.nlm.nih.gov/SNP).

### Cell culture and reverse transcription-polymerase chain reaction (RT-PCR)

**Figure 2:**
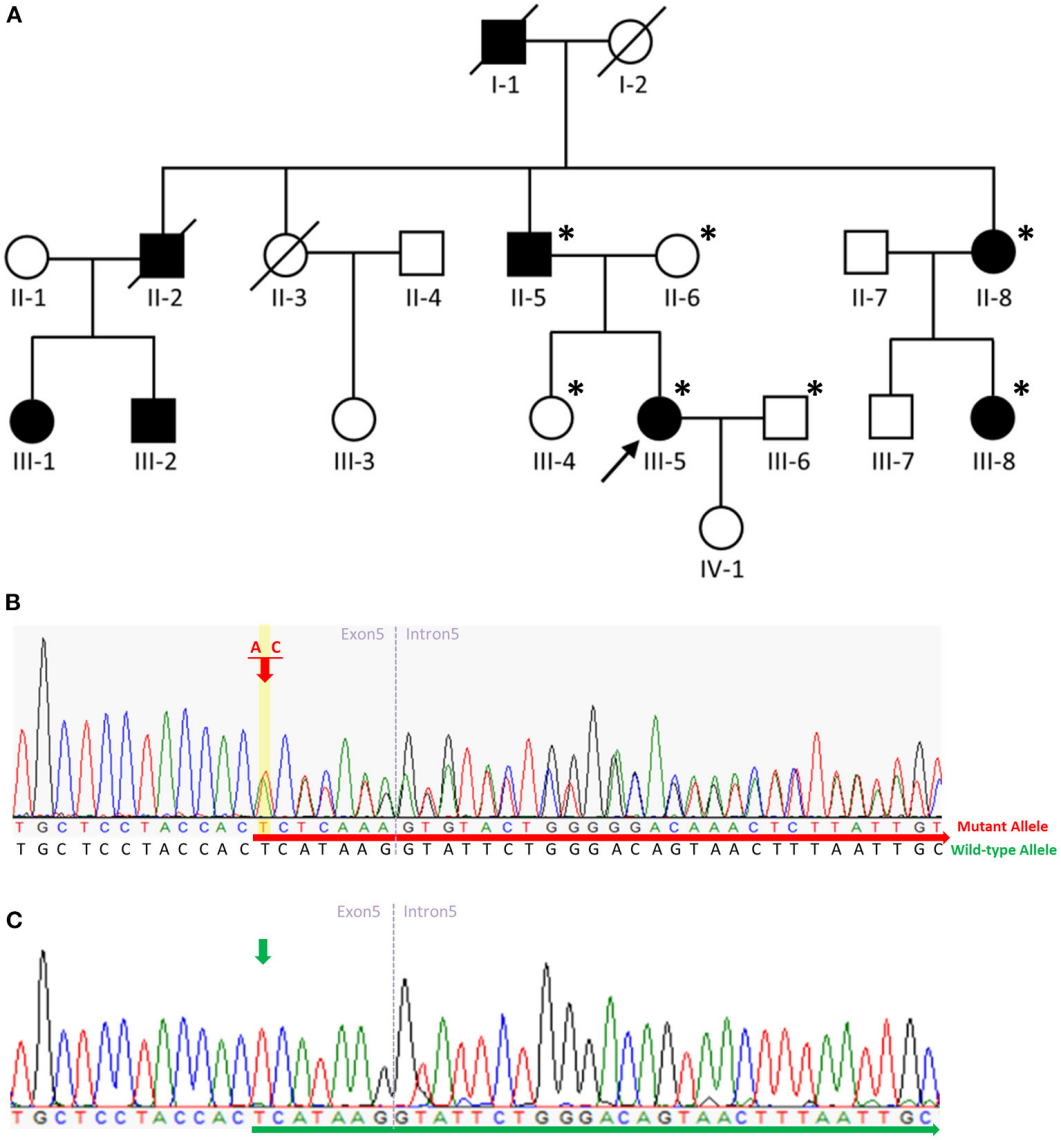
Pedigree of the SPG4 family and sequence analysis. **(A)** The proband is indicated (arrow). Squares indicate males; circles, females; slashes, deceased individuals; black symbols, individuals with symptoms of HSP; and unshaded symbols, individuals without symptoms of HSP. Patients evaluated clinically and genetically are each denoted by an asterisk. **(B)** Sequence analysis revealed a c.862_863dupAC frameshift variant in exon 5 of *SPAST* in the proband, her father, aunt, and aunt's daughter. The red arrow indicates the c.864 nucleotide. **(C)** Sequence analysis revealed no variant in exon 5 of *SPAST* in the proband's mother, husband, or sister. The green arrow indicates the c.864 nucleotide.

We performed RT-PCR, Sanger sequencing, and quantitative RT-PCR using total RNA from an Epstein-Barr virus (EBV)-induced lymphoblastoid cell line (LCL) of the proband. Mononuclear cells from the patient's peripheral blood were immortalized using the concentrated EBV supernatant as previously described (10). Total RNA from cultured lymphoblastoid cells from the proband (Figure 2A, III-5) was extracted using Trizol (Invitrogen) according to the manufacturer's instructions. Reverse transcription was performed using a Bio-Rad's iScript™ gDNA Clear cDNA Synthesis Kit (Bio-Rad Laboratories, Hercules, CA). Complementary DNA was then amplified by long-range PCR using a primer set designed to amplify from exon 1 to exon 17 of spastin M87 isoform. Quantitative RT-PCR (qRT-PCR) was performed with SsoAdvanced™ Universal SYBR^®^ Green Supermix (Bio-Rad). Normalization and relative quantification of the expression levels of the *SPAST* gene was performed using the ΔCT method, with constitutively expressed gene *18SRNA* as an internal control. The primer sequences used in this study were listed in Supplementary Table 1.

### Immunoblotting

Cultured lymphoblastoid cells from the proband and a 58-year-old wild-type control unrelated to this family were harvested and lysed in lysis buffer (20 mM Tris-HCl, pH 7.4, 150 mM NaCl, 1 mM EDTA, 0.5% Triton X-100, and protease inhibitors). Equal amounts of protein extracts (20μg/lane) were subjected to SDS-PAGE. After SDS-PAGE, proteins were transferred onto the PVDF membrane and then immunoblotted using an anti-spastin rabbit polyclonal antibody (#PA5-53581), which could recognize both the M1 and M87 spastin isoforms (Immunogen sequence region: amino acids 234-366). To check for loading, blots were re-probed with a mouse monoclonal anti-actin antibody (#A3854). The proteins were visualized with HRP-labeled secondary antibodies using a chemiluminescence detection system (Immobilon Forte, Millipore). Digital images were captured using ImageQuant LAS4000 (GE Healthcare).

## Results

### Clinical study

We describe a four-generation family with spastic paraplegia that is inherited in an autosomal dominant manner. The proband (Figure 2A, III-5), a 55-year-old female, had walked normally until age 35 when she developed gait unsteadiness and difficulty descending the staircase. Over time, her gait gradually became slow and spastic, and she experienced frequent falls. At age 48, she had to walk with the aid of crutches. A neurological examination at age 55 revealed a scissor gait, lower-limb muscle weakness, ankle clonus, and brisk bilateral knee reflexes. No muscle hypertonia or atrophy was detected, and plantar reflexes were flexor. Her cognitive functioning was normal, and no signs of cerebellar, sensory, or autonomic abnormalities were found. Studies on nerve conduction and magnetic resonance imaging of the brain and spinal cord were unremarkable.

The proband's deceased paternal grandfather (I-1), deceased uncle (II-2), father (II-5), aunt (II-8), and aunt's daughter (III-8) all presented with spastic gait. The proband's uncle's daughter (III-1) presented with spastic gait during childhood, while the proband's uncle's son (III-2) presented with spastic gait in his fifties. The proband's father (II-5) aged 84 presented with spastic gait in his early thirties. All the affected family members showed a pure form of HSP, and they could be ambulatory with crutches. The proband's 59-year-old sister (III-4) was unaffected. The clinical characteristics of the patients with the p.H289Lfs^*^27 variant in *SPAST* in this family are summarized in Supplementary Table 2.

### Genetic study

We carried out WES of genomic DNA from the proband (III-5). We examined variants of 168 genes known to be involved in Charcot-Marie-Tooth disease or HSP (Supplementary Table 3). Through this analysis, we identified a heterozygous frameshift variant (c.862_863dupAC, p. H289Lfs^*^27 [NM_014946]) in exon 5 of the *SPAST* gene in the patient and did not find any additional causal genetic variants in other genes. We then examined exon 5 of the *SPAST* gene in the proband (III-5), the proband's father (II-5), mother (II-6), sister (III-4), husband (III-6), aunt (II-8), and aunt's daughter (III-8) by Sanger sequencing. On Sanger sequencing, we found the c.862_863dupAC variant in *SPAST* in a heterozygous state in the proband, her father, aunt, and aunt's daughter (Figure 2B). This variant was not detected in the proband's mother, husband, or sister without symptoms (Figure 2C), indicating the variant co-segregated with pure HSP in this family. This variant was not present in HGMD (https://portal.biobase-international.com; Last Access Date: 2022-10-22), ClinVar (https://www.ncbi.nlm.nih.gov/clinvar/; Last Access Date: 2022-10-22), or GnomAD (http://gnomad.broadinstitute.org; Last Access Date: 2022-10-22), thus we considered it a novel molecular variant causing SPG4. Bioinformatic analyses using Mutation Taster (http://www.mutationtaster.org) predicted that this variant was disease-causing. This variant is designated as pathogenic in accordance with the criteria of the American College of Medical Genetics and Genomics (ACMG) (PVS1 + PS4 + PM2 + PP1 + PP3 + PP4) (11). It is noteworthy that the c.866_870del (p.His289fs) and c.867_868del (p.His289fs) variants were reported on ClinVar. The c.867_868del (p.His289fs) variant was also reported in the literature with **one** patient presenting a pure form of HSP and another SPG4 pedigree complicated with dysarthria (12). Including the variant we report here, all **three** truncating variants arising from the same amino acid (His289) seem to be pathogenic and causal for SPG4. Moreover, the c.843_846dup (p.Gly283fs) and c.883dup (p.Thr295fs) variants which are located nearby were also reported on ClinVar. While these **two** variants have not been reported in the literature, they were both classified as pathogenic on ClinVar.

### Transcription analysis

**Figure 3:**
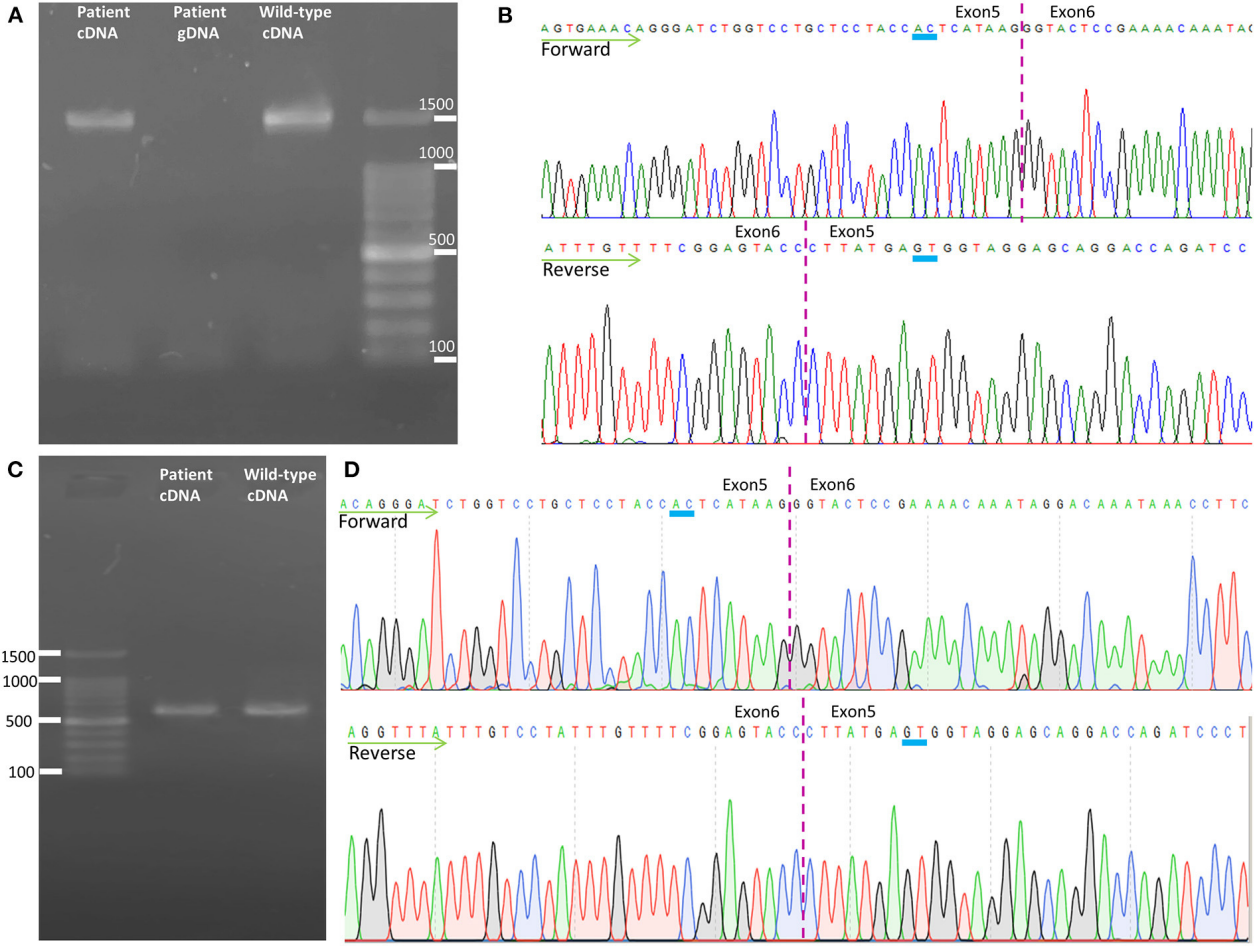
Transcription analysis of spastin in lymphoblast cell lines. **(A)** Agarose gel electrophoresis of the long-range RT-PCR products revealed only the cDNA fragment of 1,494 bp corresponding to the predicted canonical transcripts in both the patient and the control. **(B)** Sanger sequencing of long-range RT-PCR products revealed only the canonical transcript in the proband, which was reconfirmed on sequencing with a reverse primer. The transcript with the frameshift variant was not detected. **(C)** Agarose gel electrophoresis of the shorter RT-PCR products revealed only the 603 bp band expected in both the patient and control. **(D)** Sanger sequencing of the shorter RT-PCR products in both directions identified only canonical transcripts in the patient. cDNA, complementary DNA; gDNA, genomic DNA.

**Figure 4:**
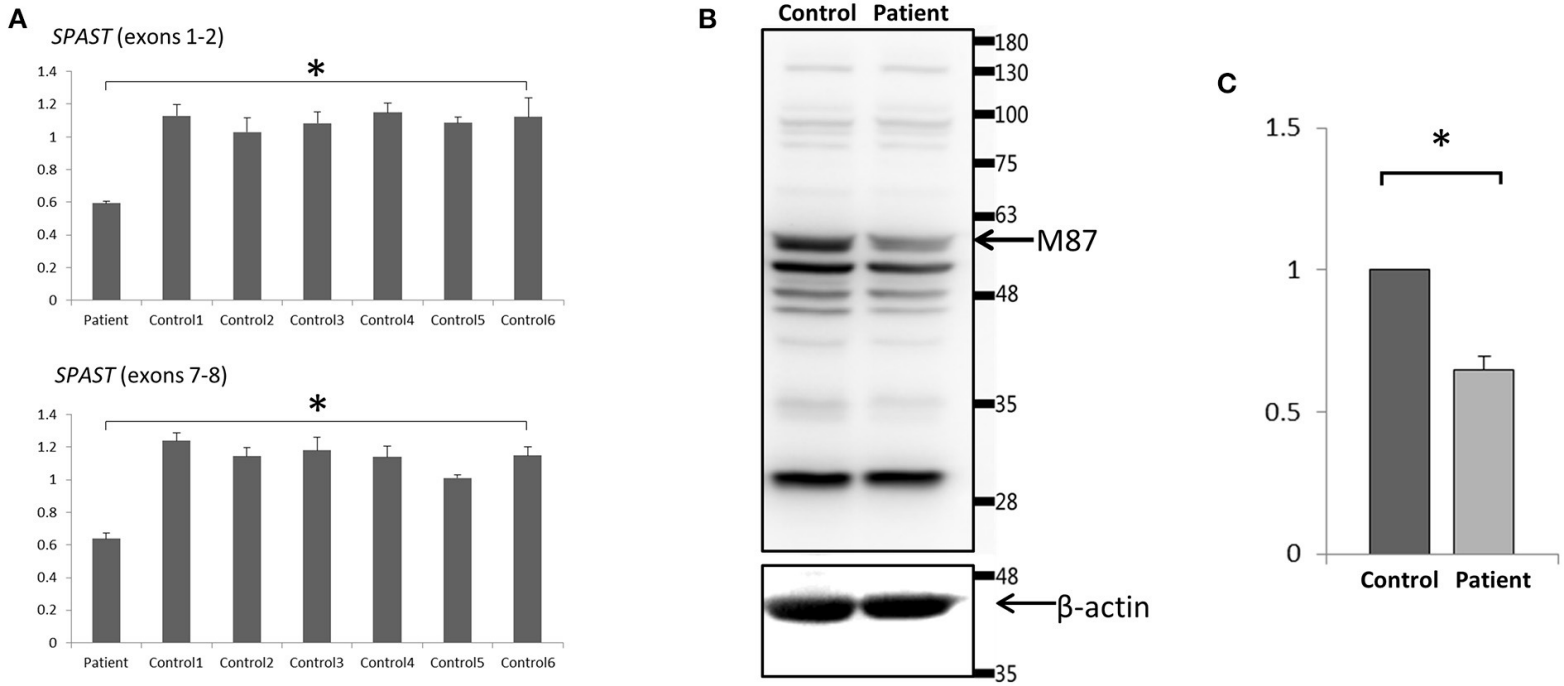
Expression analysis of spastin in lymphoblast cell lines. **(A)** Quantitative PCR of *SPAST* cRNA showed a ~50% decrease standardized against *18SRNA* in the proband compared with 6 wild-type controls. Data were the mean (±SD) of three independent experiments, Mann-Whitney U test; ^*^p < 0.01. **(B)** Western Blotting analysis revealed a decreased level of the spastin protein in the patient compared to the control. Blots are representative images from 3 independent experiments. **(C)** Densitometry analysis of the peak density normalized over β-actin showed a ~40% decrease of spastin protein in the patient compared with the control. Data were the mean (±SD) of three independent experiments, ^*^P < 0.05. The Mann-Whitney test was used to compare the two groups.

To analyze the mRNA expression affected by the variant (c.862_863dupAC, p. H289Lfs^*^27) in *SPAST*, we performed RT-PCR using total RNA from an EBV-induced lymphoblastoid cell line produced from peripheral blood mononuclear cells of the proband (10, 13). Agarose gel electrophoresis of the long-range RT-PCR products revealed only the 1,494 bp band expected in both the patient and the control, but no aberrant bands (Figure 3A). Sanger sequencing from both directions of the RT-PCR products identified only canonical transcripts in the patient (Figure 3B). The same results were obtained with a different set of primers targeting a shorter fragment (exon3-exon8). Agarose gel electrophoresis of the shorter RT-PCR products revealed only the 603 bp band expected in both the patient and control (Figure 3C). Sanger sequencing of the RT-PCR products in both directions identified only canonical transcripts in the patient (Figure 3D). Therefore, no additional spastin isoforms were found, making alternative splicing less likely. Moreover, the truncated transcript was not detected in the patient. We then examined mRNA levels in the proband's lymphoblasts by qRT-PCR. The results showed that the expression of *SPAST* transcripts was decreased by approximately 0.5-fold in the patient compared to the **six** wild-type controls when normalized to the *18SRNA* levels (Figure 4A).

### Protein expression analysis

To investigate the protein expression of spastin affected by this variant, Western blot analysis of spastin was performed on cultured lymphoblastoid cell lysates from the proband and wild-type control. The band that would correspond to the wild-type spastin M87 protein (60 kD) [NP_055761] was detected, however, the spastin protein level was decreased in the patient (~40%) compared to the control (Figures 4B,C). Therefore, we may conclude that the mutant *SPAST* transcript harboring the premature termination codon (p.H289Lfs^*^27) was subjected to nonsense-mediated mRNA decay pathway and degraded. The band that would correspond to the wild-type spastin M1 protein (68 kDa) was not detected (Figure 4B), nor was it detected by immunoblotting using a spastin antibody raised specifically against the N-terminal region of the M1 spastin isoform (data not shown).

## Discussion

We report an SPG4 family with a novel heterozygous frameshift pathogenic variant in *SPAST*. No complicated HSP-related clinical signs were noticed in any affected family members, and the disease in this pedigree was apparently autosomal dominant uncomplicated HSP. All the affected family members presented with a relatively mild phenotype, but they showed somehow different ages of onset, from childhood to over fifty years old.

All types of mutations have been described in *SPAST*. Missense mutations are primarily found in the AAA domain, but nonsense, splice-site, and insertion/deletion mutations can be found throughout the gene in various regions (Figure 1) (14). Together with the variant we report, **three** truncating variants arising from the same amino acid (His289) have been reported, indicating a possible mutation hotspot in *SPAST*. However, functional studies were not performed on c.866_870del (p.His289fs) and c.867_868del (p.His289fs) variants identified in SPG4 patients. Our study may provide preliminary functional evidence that all **three** *SPAST* gene variants are probably pathogenic.

Two start codons found in *SPAST* mRNA control the synthesis of **two** different spastin isoforms, M1 (68 kDa) and M87 (60 kDa) (15). Both isoforms also have low levels of splicing variants (16). In our study, only wild-type M87 (60 kDa) spastin isoform was detected by Western blot analysis. The M1 isoform was not detectable. Our results are consistent with previous studies that M87 predominates in all tissues and at all developmental stages, whereas M1 is mostly detectable in nerve tissue (15, 17). Although we cannot be certain that the observed decrease in spastin protein in lymphoblasts is representative of the brain or spinal cord where the M1 isoform of spastin is more abundant than in other tissues, our study may reveal the transcription and translation characteristics of the p.H289Lfs^*^27 variant in *SPAST* in this patient.

To date, different mechanistic hypotheses for the etiology of truncating variants in *SPAST*-HSP have been proposed. We have summarized the pathogenic truncating variants that have had transcription or protein expression analysis performed in previous studies (Supplementary Table 4) (16, 18–26). Haploinsufficiency has been the explanation for truncating *SPAST* mutations in the majority of the literature. The majority of mRNAs with premature termination codons in *SPAST* degrade rapidly. Meanwhile, western blot analysis of lymphoblastoid cell lines, fibroblasts, olfactory neurosphere-derived cells, and pluripotent stem cells produced from fibroblasts of the SPG4 patients revealed a decrease in the wild-type spastin protein and the absence of detectable quantities of truncated spastins (16, 19, 21–26). However, some truncating variants (c.550dupT, c.734C > G, and c.985dupA) escape degradation and are stable. Their truncated mutant proteins could accumulate to a higher level than their wild-type counterparts, indicating possible gain-of-function mechanisms (18, 20). The authors concluded that premature stop codons caused by *SPAST* mutations do not always result in haploinsufficiency (18). They hypothesized that the position of the premature termination codon from an exon-exon junction in *SPAST* may be crucial to preventing nonsense-mediated mRNA decay. Moreover, the less stable mRNAs with premature termination codons might be translated into truncated spastin proteins with greater stability (18, 20). Therefore, it would be important to perform transcription and protein expression analysis for different truncating *SPAST* pathogenic variants, as these studies would provide insight into the mechanisms underlying their pathogenicity.

## Conclusion

We report a novel frameshift pathogenic variant (p. H289Lfs^*^27) in *SPAST* from a four-generation family with a phenotype of pure HSP. The truncating variant in *SPAST* resulted in mRNA instability and reduced protein expression levels. Our study implies haploinsufficiency as the pathogenic mechanism for this variant and expands the known mutation spectrum of *SPAST*.

## Data availability statement

The datasets presented in this study can be found in online repositories. The name of the repository and accession number can be found below: [GenBank: BankIt2579948 Seq1 ON453993], https://www.ncbi.nlm.nih.gov/nuccore/ON453993.

## Ethics statement

The studies involving human participants were reviewed and approved by Institutional Review Board of Capital Medical University. The patients/participants provided their written informed consent to participate in this study. Written informed consent was obtained from the individual(s) for the publication of any potentially identifiable images or data included in this article.

## Author contributions

HN conceived the present idea, collected and analyzed the data, and wrote the manuscript. MC, LL, and KX participated in data collection. LW contributed to the conception and revision of the manuscript. All authors contributed to the article and approved the submitted version.

## Funding

This work was supported by Beijing Municipal Science and Technology Committee (No.7202060) and National Natural Science Foundation of China (No.81971011).

## Conflict of interest

The authors declare that the research was conducted in the absence of any commercial or financial relationships that could be construed as a potential conflict of interest.

## Publisher's note

All claims expressed in this article are solely those of the authors and do not necessarily represent those of their affiliated organizations, or those of the publisher, the editors and the reviewers. Any product that may be evaluated in this article, or claim that may be made by its manufacturer, is not guaranteed or endorsed by the publisher.
