# Supplementary material for: A novel truncating variant of SPAST associated with hereditary spastic paraplegia indicates a haploinsufficiency pathogenic mechanism
Source: Front Neurol. 2022 Nov 14;13:1005544. doi: 10.3389/fneur.2022.1005544 (PMC9703935; doi:10.3389/fneur.2022.1005544)
Supplement: Supplementary file 1 [file Table_1.DOCX]

Table 1. The list of primers used for cDNA PCR and qRT-PCR in this study.

| Primer name | Purpose | Forward Sequences (5’-3’) | Reverse Sequences (5’-3’) | Annealing temperature  (ºC) |
| --- | --- | --- | --- | --- |
| *SPAST* (cDNA-FL) | long-range PCR | CGTCCGAGTCTTCCACAAACAGG | TCTCCAAAGTCCTTGTTCCAACGTATG | 64 |
| *SPAST* (cDNA-exon3-8) | shorter-range PCR | GCCAAGGACCGCTTACAACTTC | ATTGTCTTCCCATTCCCAGGTG | 62 |
| *SPAST* (exons 1-2) | qRT-PCR | CGTCCGAGTCTTCCACAAACAGG | ACCATTCCACAGCTTGCTCCTTC | 63 |
| *SPAST* (exons 7-8) | qRT-PCR | GATGATATAGCTGGTCAAGACTTGGCA | ATTGTCTTCCCATTCCCAGGTG | 62 |
| *18SRNA* (internal control) | qRT-PCR | CAGCCACCCGAGATTGAGCA | TAGTAGCGACGGGCGGGTGT | 64 |

PCR: polymerase chain reaction; qRT-PCR: quantitative reverse transcription PCR; FL: full length
