# Supplementary material for: A novel truncating variant of SPAST associated with hereditary spastic paraplegia indicates a haploinsufficiency pathogenic mechanism
Source: Front Neurol. 2022 Nov 14;13:1005544. doi: 10.3389/fneur.2022.1005544 (PMC9703935; doi:10.3389/fneur.2022.1005544)
Supplement: Supplementary file 2 [file Table_2.docx]

Supplementary Material

Supplementary Table 2. Clinical characteristics of SPG4 patients with the p.H289Lfs*27 mutation in *SPAST* in this family.

| Patient | I-1 | II-2 | II-5 | II-8 | III-1 | III-2 | III-5 | III-8 |
| --- | --- | --- | --- | --- | --- | --- | --- | --- |
| Age at onset | Late 30s | childhood | 31 | 45 | 11 | 52 | 35 | 29 |
| Clinical phenotype | pure form | pure form | pure form | pure form | pure form | pure form | pure form | pure form |
| Babinski sign | unknown | unknown | + | + | + | − | − | − |
| Sensory disturbance | unknown | unknown | − | − | − | − | − | − |
| Autonomic disorder | − | − | − | − | − | − | − | − |
| Others | − | died of cerebral ischemic stroke | − | senile cataract | − | finger hyperextension | − | − |
| ADL | unknown | ambulatory with two canes | ambulatory with a cane | unassisted ambulatory | ambulatory with a cane | unassisted ambulatory | ambulatory with two canes | unassisted ambulatory |
