# Supplementary material for: A novel truncating variant of SPAST associated with hereditary spastic paraplegia indicates a haploinsufficiency pathogenic mechanism
Source: Front Neurol. 2022 Nov 14;13:1005544. doi: 10.3389/fneur.2022.1005544 (PMC9703935; doi:10.3389/fneur.2022.1005544)
Supplement: Supplementary file 3 [file Table_3.DOCX]

Supplementary Material

Supplementary Table 3. Genes known to be responsible for Charcot-Marie-Tooth disease and HSP.

*ATL1 SPAST NIPA1 KIAA0196 ALDH18A1 KIF5A RTN2 HSPD1 BSCL2 ATSV REEP1 ZFYVE27 SLC33A1 REEP2 CPT1C CYP7B1 SPG7 SPG11 ZFYVE26 ERLIN2 SPG20 ACP33 B4GALNT1 DDHD1 FA2H PNPLA6 c9orf12 GJA12 NT5C2 GBA2 AP4B1 KIAA0415 TECPR2 AP4M1 AP4E1 AP4S1 VPS37A DDHD2 c12orf65 CYP2U1 TFG KIF1C USP8 WDR48 ARL6IP1 ERLIN1 AMPD2 ENTPD1 ARSI PGAP1 FLRT1 RAB3GAP2 MARS ZFR IBA57 MAG MTCO3 MTTI MTND4 MTATP6 L1CAM PLP Xq11 SLC16A2 Xp25 SPPRS BICD2 CHS IFIH1 CCT5 FAM134B ALS2 EXOSC3 SPOAN GAD1 ARSACS HACE1 LYST SACS AARS ABHD12 AIFM1 ARHGEF10 ARSA ASAH1 COX6A1 CTDP1 DCAF8 DGAT2 DHH DHT DNAJB2 DNAJC3 DNM2 DRP2 DYNC1H1 EGR2 EMILIN1 FBLN5 FGD4 FIG4 GALC GAN GARS GDAP GDAP1 GJB1 GJB3 GNB4 HARS HINT1 HK1 HOXD10 HSPB1 HSPB8 IFRD1 IGHMBP2 INF2 KARS KCC3 KIF1B LITAF LMNA LRSAM1 MED25 MFN2 MME MORC2 MPZ MTMR2 NAGLU NDRG1 NEFH NEFL PDK3 PEX7 PHYH PLA2G6 PLEKHG5 PMM2 PMP22 PRPS1 PRX RAB7 SBF1 SBF2 SCYL1 SH3TC2 SLC25A46 SOX10 SPTLC1 SPTLC2 SPTLC3 SURF1 TDP1 TRIM2 TRPV4 TUBB3 VCP YARS KIF1A UBAP1 SELENOI PCYT2 HPDL PI4Ka RNF170 ABHD16A*
