# Supplementary material for: A novel truncating variant of SPAST associated with hereditary spastic paraplegia indicates a haploinsufficiency pathogenic mechanism
Source: Front Neurol. 2022 Nov 14;13:1005544. doi: 10.3389/fneur.2022.1005544 (PMC9703935; doi:10.3389/fneur.2022.1005544)
Supplement: Supplementary file 4 [file Table_4.DOCX]

Supplementary Material

Supplementary Table 4. A summary of pathogenic truncating variants in *SPAST* that have had transcription or protein expression analysis performed in previous studies.

| Study (First author), the year of publication | Mutations in *SPAST* | Analyzing material and methords | mRNA nonsense-mediated decay | Proposed mechanism underlying *SPAST*-HSP |
| --- | --- | --- | --- | --- |
| Joachim Bürger, 2000 | c.1617-1618+2del | Lymphocytes | Yes | Haploinsufficiency |
| Joachim Bürger, 2000 | c.1853+1G>T | Lymphocytes | Yes | Haploinsufficiency |
| Ingrid K. Svenson, 2001 | IVS9+4A>G | Lymphocytes | Yes | Haploinsufficiency |
| Ingrid K. Svenson, 2001 | IVS11+2T | Lymphocytes | Yes | Haploinsufficiency |
| Ingrid K. Svenson, 2001 | IVS9+1G>A | Lymphocytes | Yes | Haploinsufficiency |
| Ingrid K. Svenson, 2001 | IVS12−2A>C | Lymphocytes | Yes | Haploinsufficiency |
| Delphine Charvin, 2003 | Q193X | Lymphoblastoid cell lines | Yes | Haploinsufficiency |
| Delphine Charvin, 2003 | Q229X | Lymphoblastoid cell lines | Yes | Haploinsufficiency |
| Delphine Charvin, 2003 | c.1634del22 | Lymphoblastoid cell lines | Yes | Haploinsufficiency |
| Elena Riano, 2009 | c.1634C>G p.S545X | Lymphoblastoid cell lines | Yes | Haploinsufficiency |
| Elena Riano, 2009 | c.1536+1 del4 p.R498fsX17 | Lymphoblastoid cell lines | Yes | Haploinsufficiency |
| Elena Riano, 2009 | c.1413+2 T>G p.D441fsX6 | Lymphoblastoid cell lines | Yes | Haploinsufficiency |
| Elena Riano, 2009 | c.870+3A>G p.E228fsX6 (homozygous) | Lymphoblastoid cell lines | Yes | Haploinsufficiency |
| Greger Abrahamsen, 2013 | c.1413+3_1413+6del | Olfactory neurosphere-derived cells | Yes | Haploinsufficiency |
| Greger Abrahamsen, 2013 | p.W148X c.444G>A | Olfactory neurosphere-derived cells | Yes | Haploinsufficiency |
| Greger Abrahamsen, 2013 | Exons 8-9 deletion | Olfactory neurosphere-derived cells | Yes | Haploinsufficiency |
| Kyle R. Denton, 2014 | c.683-1G>T | iPSCs cells generated from dermal fibroblasts | Yes | Haploinsufficiency |
| Joanna M. Solowska, 2017 | c.550dupT p.Asn184Ter | Plasmid Construction pTRE-Tight vector | No | Toxic gain-of-function |
| Joanna M. Solowska, 2017 | c.734C > G p.Ser245Ter | Plasmid Construction pTRE-Tight vector | No | Toxic gain-of-function |
| Toshitaka Kawarai, 2017 | c.1004+3A>C p.Gly290Trpfs*5 | Lymphocytes | Yes | Haploinsufficiency |
| Timothy Newton, 2018 | *SPAST* deletion involving exons 2-9 | Fibroblasts | Yes | Haploinsufficiency |
| Rui Chen, 2022 | c.985dupA p.Met329Asnfs*3 | Plasmid Construction pEGFP-C1 expression vector | No | Toxic gain-of-function |
